# miRNA-205 targets VEGFA and FGF2 and regulates resistance to chemotherapeutics in breast cancer

**DOI:** 10.1038/cddis.2016.194

**Published:** 2016-06-30

**Authors:** Y Hu, Y Qiu, E Yagüe, W Ji, J Liu, J Zhang

**Affiliations:** 1 The Third Department of Breast Cancer, China Tianjin Breast Cancer Prevention, Treatment and Research Center, Tianjin Medical University Cancer Institute and Hospital, National Clinical Research Center of Cancer, Huan-Hu-Xi Road, Ti-Yuan-Bei, He Xi District, Tianjin 300060, PR China; 2 Key Laboratory of Breast Cancer Prevention and Therapy of Ministry of Education, Huan-Hu-Xi Road, Ti-Yuan-Bei, He Xi District, Tianjin 300060, PR China; 3 Cancer Research Center, Division of Cancer, Faculty of Medicine, Imperial College London, Hammersmith Hospital Campus, London W12 0NN, UK

## Abstract

MicroRNAs (miRNAs) have critical roles in regulating cancer cell survival, proliferation and sensitivity to chemotherapy. The potential application of using miRNAs to predict chemotherapeutic response to cancer treatment is highly promising. However, the underlying mechanisms of chemotherapy response control by miRNAs remain to be fully identified and their prognostic value has not been fully evaluated. Here we show a strong correlation between miR-205 expression and chemosensitivtiy to TAC (docetaxol, doxorubicin plus cyclophosphamide), a widely-used neoadjuvant chemotherapy (NAC) regimen, for breast cancer patients. High level of miR-205 predicted better response to TAC regimen NAC in breast cancer patients. We found miR-205 downregulated in both MCF-7/A02 and CALDOX cells, two drug-resistant derivatives of MCF-7 and Cal51 cells, and its ectopic expression led to an increase in apoptosis resensitization of both drug-resistant cell lines to doxorubicin and taxol. We further show that miR-205 directly binds *VEGFA* and *FGF2* mRNA 3′-UTRs and confirm that miR-205 levels are negatively correlated with *VEGFA* and *FGF2* mRNA expression in breast cancer patients. Adding VEGFA and FGF2 exogenously to chemosensitive breast cancer cells and chemoresistant cells with miR-205 overexpression led to drug resistance. Consistently, low VEGFA and FGF2 expression correlated with better response to NAC in breast cancer patients. In addition, inhibition of tumor growth and resensitization to doxorubicin were also observed in mouse tumor xenografts from cells overexpressing miR-205. Taken together, our data suggest that miR-205 enhances chemosensitivity of breast cancer cells to TAC chemotherapy by suppressing both VEGFA and FGF2, leading to evasion of apoptosis. MiR-205 may serve as a predictive biomarker and a potential therapeutic target in breast cancer treatment.

Despite the progress in early diagnosis, breast cancer remains the most common cancer in women worldwide.^1^ Neoadjuvant chemotherapy (NAC) is one of the most crucial factors for tumor burden reduction and successful breast-conserving surgery. In addition, using chemotherapy in the neoadjuvant setting allows monitoring *in vivo* tumor response to chemotherapeutics,^2, 3^ and analyzing residual disease after NAC may reveal novel therapeutic targets.^4^

Generally, only a fraction of breast cancer patients achieve full response to NAC.^5, 6^ Unfortunately, there is no reliable method for predicting chemotherapeutic responders from non-responders,^7^and there is an urgent need to stratify these patients in order to avoid unnecessary chemotherapy side effects. Recent efforts have focused on the characterization of biomarkers able to predict response to NAC, with the aim to tailor patient-care programs, reduce chemotherapy-induced morbidity or mortality and identify novel targets to be used in the development of innovative and more efficient therapies for the treatment of breast carcinoma.

MicroRNAs (miRNAs), a class of highly conserved, short, non-protein-coding RNAs that negatively regulate gene expression, have emerged as crucial regulators of the drug response by modulating dug efflux, cell apoptosis, epithelial–mesenchymal transition (EMT) and cancer stem cells.^8, 9, 10^ Previous studies have revealed that numerous miRNAs are upregulated or downregulated in breast cancer, contributing to the initiation and development of the disease, as well as its drug sensitivity.^11, 12, 13^ For instance, overexpression of miRNA-451 sensitizes breast cancer cells to doxorubicin,^14^ and upregulation of miRNA-21 is associated with acquired trastuzumab resistance.^15^ Moreover, we have recently reported that the miR-106b-93-25 cluster leads to activation of EMT transition and resistance to doxorubicin and taxol.^16, 17^ However, predictive miRNA signatures of NAC response remain to be found and fully validated.

We previously reported that miR-205 may function as a tumor suppressor, as its expression is reduced in breast tumors. Importantly, experimental restoration of miR-205 expression in breast cancer cells inhibits cell proliferation and promotes apoptosis.^18^ In this study, we show that high levels of miR-205 predict sensitivity to TAC (docetaxol, doxorubicin and cyclophosphamide) regimen in breast cancer patients. MiR-205 is downregulated in drug-resistant derivates of MCF-7 and Cal51 cells and its ectopic expression resensitizes both drug-resistant cells to doxorubicin and taxol. We demonstrate that miR-205 targets vascular endothelial growth factor A (VEGFA) and fibroblast grow factor-2 (FGF2), resulting in decreased phosphatidylinositol 3-kinase (PI3K)/Akt signaling pathway activity and increased apoptosis upon chemotherapy. Therefore, miR-205 may be used as a predictive biomarker for TAC regimen and a potential therapeutic target in breast cancer treatment.

## Results

### miR-205 expression levels correlate with NAC response in breast cancer patients

**Figure 1:**
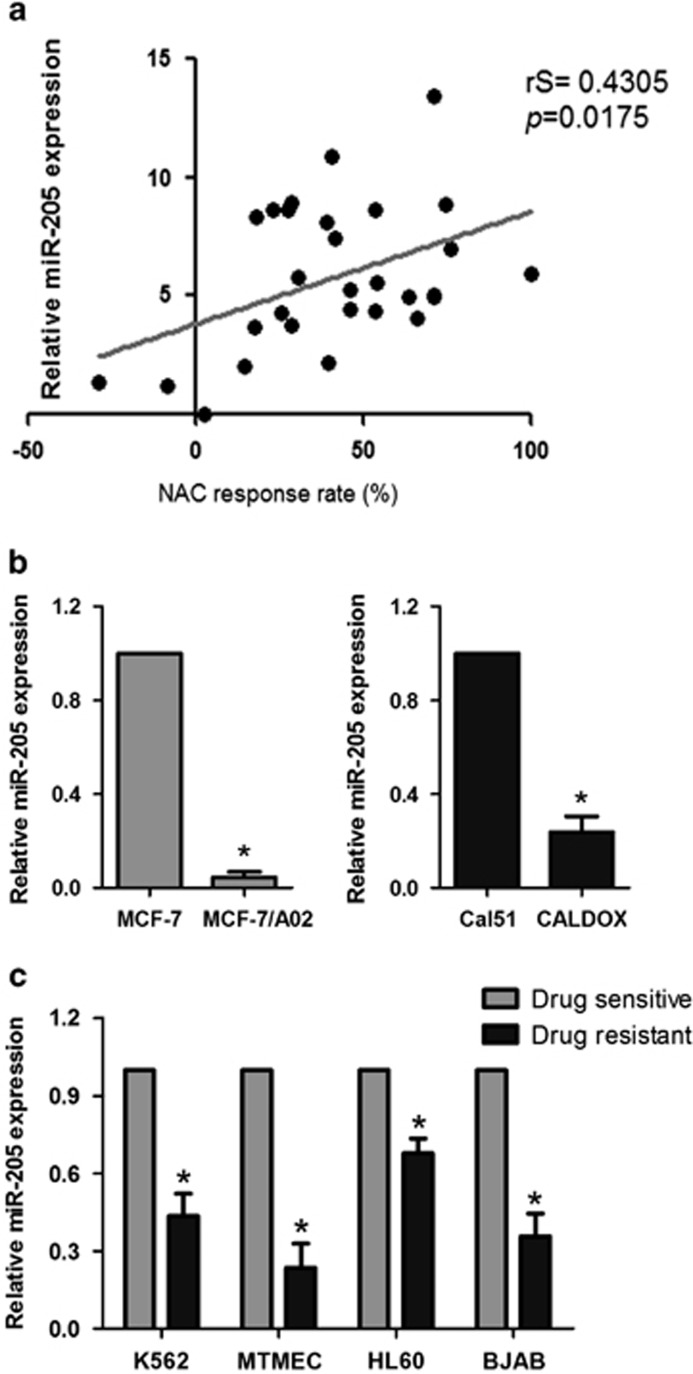
High expression of miR-205 correlates with better chemotherapeutic response. (**a**) Expression levels of miR-205 in breast cancer patients (*n*=30) who received TAC regimen NAC were determined by qPCR. Spearman rank test was used for the correlation between miR-205 and NAC response rate determined as (1−greatest tumor diameter after NAC/greatest tumor diameter before NAC) × 100%. The trend of the correlation is indicated with a line calculated by linear regression. (**b**) miR-205 expression in pairs of drug-sensitive and -resistant MCF-7 (left panel) and Cal51 (right panel) cells determined by qPCR. (**c**) miR-205 expression in pairs of drug-sensitive and -resistant K562, MTMEC, HL60 and BJAB cells. miR expression determined by qPCR was normalized to that of RNU6 RNA. Data represent the average±S.D. of three independent experiments (*P<0.05)

In order to investigate the correlation of miR-205 expression with NAC response, we collected 30 breast cancer tissue samples from patients before receiving TAC (Table 1), an anthracycline and taxane-based regimen widely used as neoadjuvant treatment of breast cancer.^19^ TAC (docetaxol, 75 mg/m^2^, doxorubicin, 50 mg/m^2^ and cyclophosphamide, 500 mg/m^2^) was administered every 3 weeks for 6 cycles as NAC. Standard RECIST guidelines were used to evaluate clinical and pathological response. We performed a linear regression and Spearman rank test and obtained a positive correlation between miR-205 expression, detected by reverse transcription quantitative real-time PCR (qRT-PCR) and NAC response rate, calculated by the alterations in greatest tumor diameter (*r*s=0.4305, *P*=0.0175) (Figure 1a). Thus miR-205 expression correlates with NAC response.

### miR-205 is downregulated in drug-resistant breast cancer cells

To confirm the positive correlation between miR-205 and TAC response shown in breast cancer patients, we further analyzed its expression in two pairs of chemosensitive and chemoresistant breast cancer cell lines. MCF-7/A02 and CALDOX have been derived from chemosensitive MCF-7 and Cal51 cells, respectively,^20, 21^ and show a traditional multidrug-resistant phenotype with cross-resistance to a wide range of structurally and functionally unrelated drugs (Supplementary Tables S1 and S2). We found miR-205 downregulated in both MCF-7/A02 and CALDOX cells by approximately 90 and 75%, respectively (Figure 1b). Similar results were also observed in other pairs of sensitive/resistant cancer cell lines such as those derived from K562, MTMEC, HL60 and BJAB cells^17, 21, 22^ (Figure 1c), although only breast cancer drug-resistant MTMECs showed similar diminished miR-205 levels as those found in the other breast cancer cell lines (Figure 1b). Thus miR-205 is downregulated in multidrug-resistant cell lines.

### Overexpression of miR-205 restores drug sensitivity by promoting apoptosis in breast cancer cells

**Figure 2:**
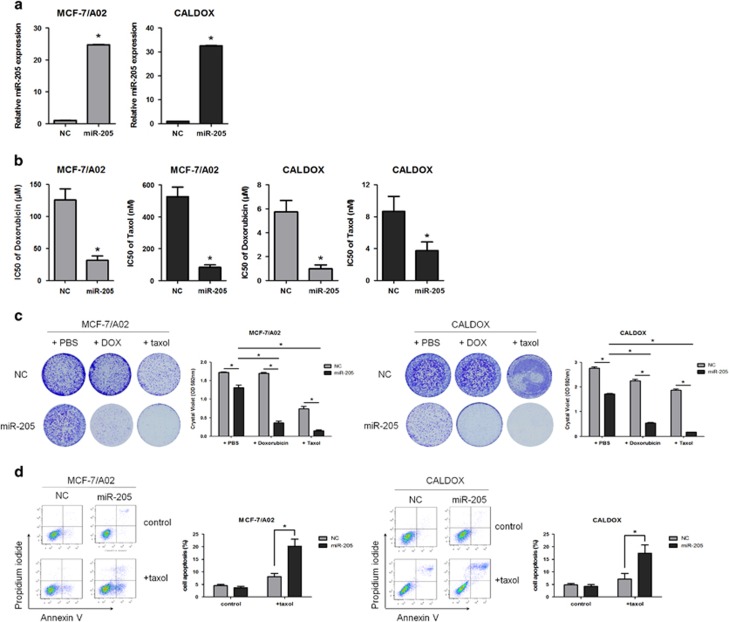

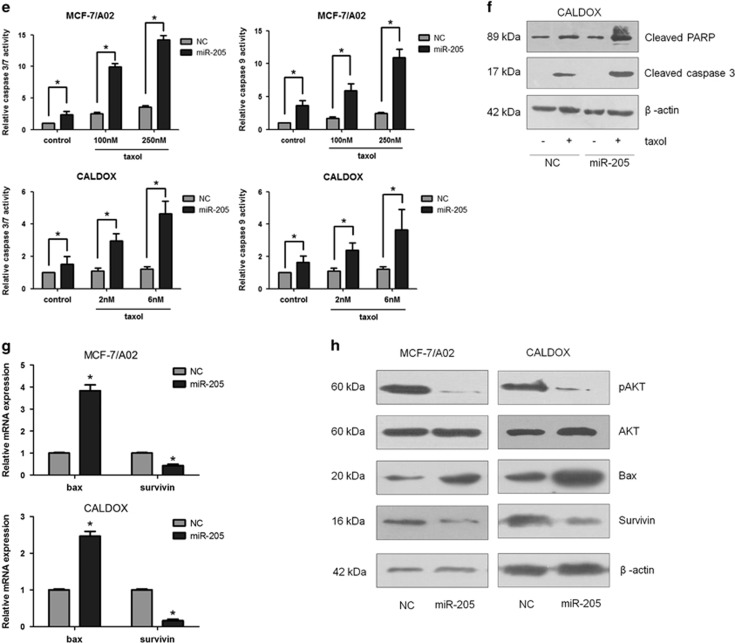
miR-205 overexpression restores drug sensitivity and promotes apoptosis in breast cancer cells. Drug-resistant MCF-7/A02 and CALDOX cells were stably transfected with a lentivirus carrying an expression vector for miR-205, generating MCF-7/A02-miR-205 and CALDOX-miR-205 cells, respectively. Control cells, MCF-7/A02-NC and CALDOX-NC were transfected with the empty vector (pGLV2-U6-Puro). (**a**) Expression of miR-205 in transfected cells determined by qPCR and normalized to RNU6 expression. (**b**) IC_50_ values of doxorubicin and taxol (3-day treatments) in transfected cells. (**c**) Effect of miR-205 on drug resistance. Cells were treated with doxorubicin (15 *μ*M for MCF-7/A02 and 1 *μ*M for CALDOX) and taxol (0.25 *μ*M for MCF-7/A02 and 2.5 nM for CALDOX) for 7 days, and the cells were stained with crystal violet. Dye was solubilized and the optical density at 592 nm was measured. (**d**) Transfected drug-resistant cells were treated with taxol (0.25 *μ*M for MCF-7/A02 and 2.5 nM for CALDOX) for 48 h. Annexin V/propidium iodide staining was detected by flow cytometry. Representative plots of three independent experiments are shown, left panels. Quantitative data show the average percentage of annexin V-positive cells (both in early apoptosis, lower right quadrant, and late apoptosis, upper right quadrant) of three independent experiments, right panels. (**e**) Caspase-3/7 and caspase-9 activities in miR-205-transfected MCF-7/A02 (upper histograms) and CALDOX cells (lower histograms) after 48-h taxol treatment. (**f**) Expression levels of cleaved PARP and cleaved caspase-3 in drug-resistant CALDOX cells were determined by western blotting after 2.5 nM taxol treatment for 48 h. (**g**) Expression levels of Bax and survivin in mir-205-transfected drug-resistant cells determined by qPCR and normalized to RPS14 expression. (**h**) Expression levels of pAKT, AKT, Bax and survivin in miR-205-transfected drug-resistant cells determined by western blotting. Numerical data represent mean±S.D. based on three independent experiments (*P<0.05). Pictorial data show representatives of three independent experiments

As miR-205 is downregulated in drug-resistant breast cancer cell lines, we hypothesized that, if it would contribute to the drug-resistant phenotype, its ectopic expression might restore drug sensitivity. To test this hypothesis, we generated stable MCF-7/A02 and CALDOX cells overexpressing miR-205 by lentiviral transfection (MCF-7/A02-miR-205 and CALDOX-miR-205, respectively). Cells transfected with empty vector (MCF-7/A02-NC and CALODX-NC) were used as control. MiR-205 expression was upregulated 24-fold in MCF-7/A02-miR-205 cells and 32-fold in CALDOX-miR-205 cells (Figure 2a). Drug sensitivity assays indicated that the doxorubicin IC_50_ values of miR-205 overexpressing cells decreased between 74 and 82% (74.4% in MCF-7/A02-miR-205 cells and 81.9% in CALDOX-miR-205 cells). Equally, miR-205-overexpressing cells were more sensitive to taxol, and the IC_50_ values of miR-205-overexpressing cells decreased between 56 and 84% (84.2% in MCF-7/A02-miR-205 cells and 56.8% in CALDOX-miR-205 cells; Figure 2b and Supplementary Figure S1). Moreover, to evaluate the efficacy of miR-205 overexpression on drug resistance, defined as proliferation in the presence of toxic drug concentrations, we stained cell cultures with crystal violet, as a surrogate for cell mass,^23^ after 1 week treatment (drug concentrations and time points were determined empirically in a series of preliminary experiments). MiR-205 restoration decreased cell proliferation in the absence of drug by 24–37% (Figure 2c), and cell cycle analysis showed a significant arrest and accumulation of cells in the G0/G1 phase (Supplementary Figure S1B), which is consistent with data in human osteosarcoma^24^ and thyoid carcinoma.^25^ Moreover, miR-205 impaired proliferation to a much higher extent in the presence of doxorubicin or taxol (reduction between 75 and 91% Figure 2c). Thus overexpression of miR-205 restores sensitivity to doxorubicin and taxol in drug-resistant breast cancer cells.

### miR-205 overexpression promotes cell apoptosis, induces caspase activity and modulates apoptosis-related proteins

Next we asked whether the drug sensitivity rescue by miR-205 on chemoresistant breast cancer cells was mediated by promoting cell apoptosis. For this, we used taxol, a widely used microtubule poison and activator of the apoptotic cascade.^26^ Annexin V staining indicated that in both drug-resistant cells ectopic expression of miR-205 increased the percentage of cells undergoing apoptosis after taxol treatment (Figure 2d). As CALDOX cells are more sensitive to taxol than MCF-7/A02 cells, the concentration of taxol used was 0.25 *μ*M for MCF-7/A02 and 2.5 nM for CALDOX cells. Approximately 20% of MCF-7/A02-miR-205 cells were in the early stages of apoptosis (positive annexin V and negative propidium iodide), whereas approximately 18% of CALDOX-miR-205 cells were already in the late stage of apoptosis (positive for both annexin V and propidium iodide) after 48-h taxol treatment (Figure 2d).

As caspases are key mediators of apoptosis,^27^ we analyzed the activation of both initiator and effector caspases in miR-205-overexpressing cells in response to taxol treatment. Both caspase 9 and caspase 3/7 activities increased between 2- and 4.5-fold after taxol treatment in drug-resistant cells overexpressing miR-205 when compared with the control cells (Figure 2e). It is also noteworthy that miR-205 overexpression increased caspase activity in both drug-free cell lines, although the activation due to drug treatment was much higher (Figure 2e). Caspase 3, the effector caspase in the apoptotic cascade, is activated through cleavage by caspase-9 as a result of increased mitochondrial permeability and release of cytochrome *c*. PARP is cleaved by activated caspase-3 and cleavage of caspase-3 and PARP are signature events in apoptosis.^28^ Consistent with the increased apoptosis in the taxol-treated miR-205-overexpressing cells, we observed a marked increase in the levels of cleavage of caspase-3 and PARP in CALDOX-miR-205 cells, especially after taxol treatment (Figure 2f). As MCF-7 cells lack caspase 3, these activities were not measured in MCF-7/A02 cells.^29^

The induction of apoptotic cell death alters the ratios of prosurvival and proapoptotic proteins. Hence, we evaluated the expression level of survivin, a survival gene, and proapoptotic Bax. RT-qPCR and western blotting results revealed that overexpressing miR-205 repressed survivin and promoted Bax expression in drug-resistant cells (Figures 2g and h). The PI3K/AKT signaling pathway is frequently dysregulated in human cancer and has been implicated in chemoresistance.^30, 31^ We also observed that the induction of cell apoptosis by miR-205 overexpression in both drug-resistant cells was associated with a significant reduction in AKT phosphorylation, suggesting a decrease in PI3K activity (Figure 2h). Thus overexpression of miR-205 in drug-resistant cells promotes apoptosis by upregulation of Bax and downregulation of survivin and the AKT pathway.

### miR-205 directly targets VEGFA and FGF2 and negatively regulates their expression

miR-205 has recently been reported to increase radiosensitivity and enhance EMT via targeting ZEB1 in breast cancer SUM159, MCF-7 and MDA-MB-231 cell lines.^32, 33^ However, we did not observe any regulation of ZEB-1 upon expression of miR-205 in either drug-resistant MCF-7/A02 or CALDOX cells (Supplementary Figures S2A and B). Moreover, we also compared ZEB-1 expression in both pairs of naive and drug-resistant derivatives. Although ZEB-1 expression was highly upregulated in MCF-7/A02 with respect to MCF-7 cells, ZEB-1 levels were not significantly elevated in CALDOX cells (Supplementary Figures S2A and B). Thus the lack of correlation between ZEB-1 and miR-205 expression levels suggests that the restoration of chemosensitivity by miR-205 in MCF-7/A02 and CALDOX cells is ZEB-1 independent.

**Figure 3:**
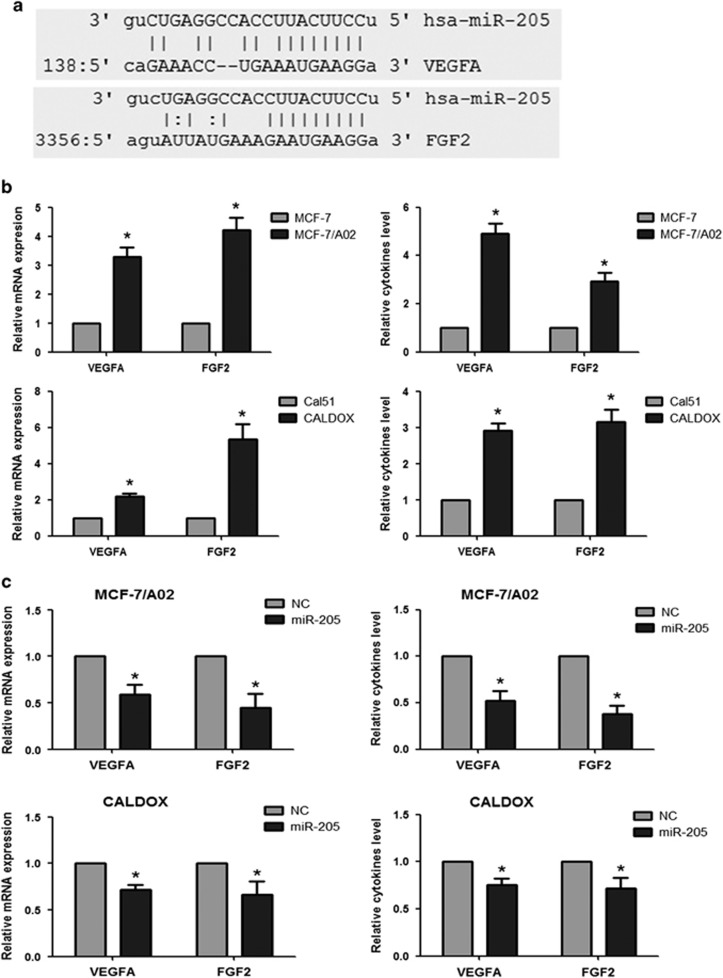

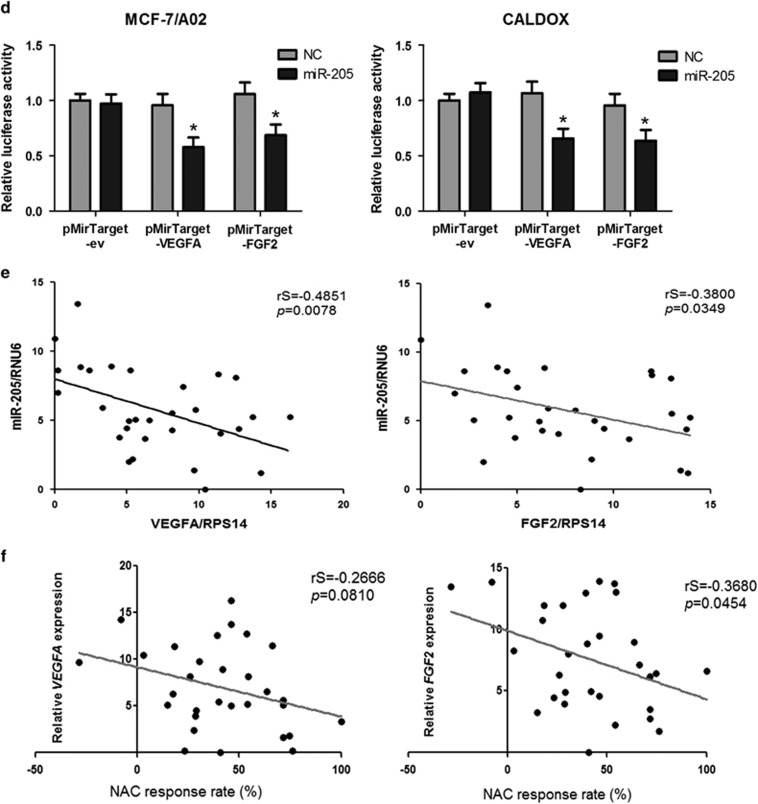
Identification of *VEGFA* and *FGF2* mRNAs as direct targets of miR-205 in breast cancer cells. (**a**) Annealing of miR-205 to *VEGFA* and *FGF2* mRNA 3′-UTRs according to the microRNA.org database. (**b**) VEGFA and FGF2 expression at mRNA (qPCR; left panels) and cytokine levels (ELISA; right panels) in drug-sensitive and -resistant breast cancer cells. (**c**) Expression levels of VEGFA and FGF2 in drug-resistant cells after stable expression of miR-205 or empty vector control (*NC*) at mRNA (qPCR; left panels) and protein levels (ELISA; right panels). (**d**) miR-205 targets *VEGFA* and *FGF2* mRNAs. Normalized firefly luciferase activity from the reporter with the *VEGFA* 3′-UTR (*pMirTarget-VEGFA*), *FGF2* 3′-UTR (pMirTarget-FGF2) or the empty vector (*pMirTarget-ev*) after transient transfection into cells overexpressing miR-205 or control (NC) cells. In all cases, cells were co-transfected with a *Renilla* luciferase expression vector to normalize for transfection efficiency. Numerical data represent mean±S.D. based on three independent experiments (*P<0.05). (**e**) Inverse correlation between miR-205 and VEGFA (left panel)/FGF2 (right panel) in breast cancer (*n*=30) as determined by Spearman rank test. The trend of the correlation is indicated with a line calculated by linear regression. (**f**) Expression levels of VEGFA (left panel) and FGF2 (right panel) in breast cancer patients (*n*=30) who received TAC regime NAC were quantitated by qPCR. Spearman rank test was used for depicting the correlation between mRNA and NAC response rate determined as (1−greatest tumor diameter after NAC/greatest tumor diameter before NAC) × 100%. The trend of the correlation is indicated with a line calculated by linear regression

Bioinformatic analysis indicates that both VEGFA and FGF2 are potential targets for miR-205 (Figure 3a), and this has been confirmed for VEGFA in osteosarcoma^24^, thyroid cancer^25^ and breast cancer MDA-MB-231 cells.^34^ VEGFA and FGF2 promote therapy resistance in a number of cancers^35, 36, 37, 38^ and, reassuringly, we have confirmed their overexpression both at the mRNA and protein levels in both MCF-7/A02 and CALDOX cells (Figure 3b). In addition, experimental expression of miR-205 in drug-resistant cells led to a downregulation of both VEGFA and FGF2 (Figure 3c), confirming the negative correlation between miR-205 and VEGFA/FGF2 expression.

To verify whether miR-205 is capable of regulating FGF2 and VEGFA expression via the binding sites in their 3′-UTRs (Figure 3a), each 3′-UTR containing the predicted miR-205 binding site was cloned downstream of the firefly luciferase coding region in the pMirTarget luciferase vector, resulting in pMirTarget-VEGFA and pMirTarget-FGF2, respectively. We transiently transfected cells overexpressing miR-205 with pMirTarget-VEGFA or pMirTarget-FGF2, together with a *Renilla* luciferase expressing plasmid to normalize for transfection efficiency. We hypothesized that luciferase expression would decrease in cells overexpressing miR-205 when transfected with the reporter plasmid incorporating *VEGFA* 3′-UTR or *FGF2* 3′-UTR but not in the control. Indeed, an approximately 40% reduction in luciferase expression was observed in both MCF-7/A02-mir-205 *versus* MCF-7/A02-NC and CALDOX-mir-205 *versus* CALDOX-NC when transfected with pMirTarget-VEGFA. In addition, an approximately 35% reduction in luciferase expression was also observed in both MCF-7/A02-mir-205 *versus* MCF-7/A02-NC and CALDOX-mir-205 *versus* CALDOX-NC when transfected with pMirTarget-FGF2. However, no changes in luciferase expression were observed in the same cells transfected with the pMirTarget-ev control vector (Figure 3d). Thus miR-205 binds *VEGFA* and *FGF2* mRNA 3′-UTRs and decreases their expression in breast cancer cells.

To confirm the negative correlation between the decreased VEGFA/FGF2 and enhanced miR-205 observed in breast cancer cell lines, we further analyzed the expression of VEGFA/FGF2 by quantitative PCR in the 30 patient samples that show positive correlation between miR-205 expression and response to TAC (Figure 1a). We found that both the expression of *VEGFA* and *FGF2* showed a negative correlation with miR-205 levels in these 30 patients (*r*s=−0.4851, *P*=0.0078 for VEGFA and miR-205, *r*s=−0.38, *P*=0.0349 for FGF2 and miR-205; Figure 3e). Additionally, we found that high VEGFA/FGF2 level was associated with poorer response to TAC regimen in these 30 patients, although the correlation between VEGFA and NAC response rate did not reach statistical significance, likely because of small sample size (Figure 3f). Collectively, these data suggest that the loss of miR-205 induces VEGFA/FGF2 expression, thus conferring chemoresistance in breast cancer.

### Repression of VEGFA and FGF2 in drug-resistant cells is essential for miR-205-induced restoration of chemosensitivity

**Figure 4:**
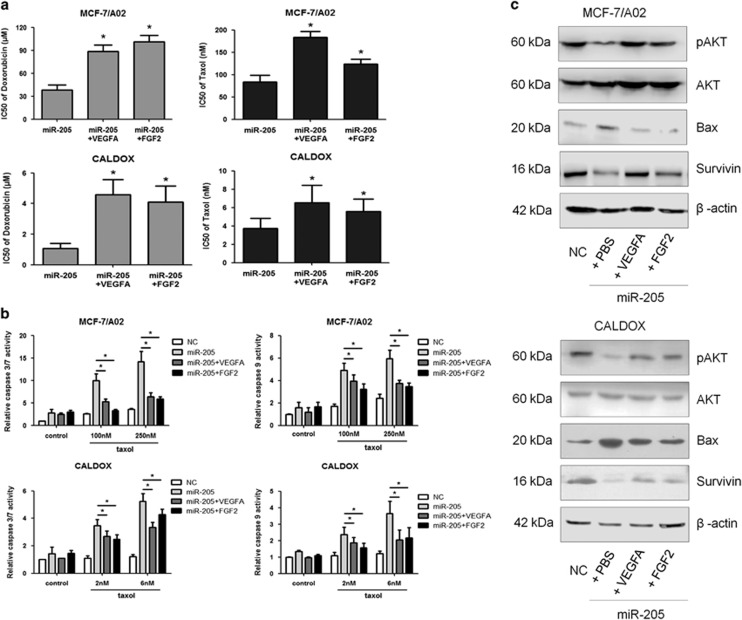

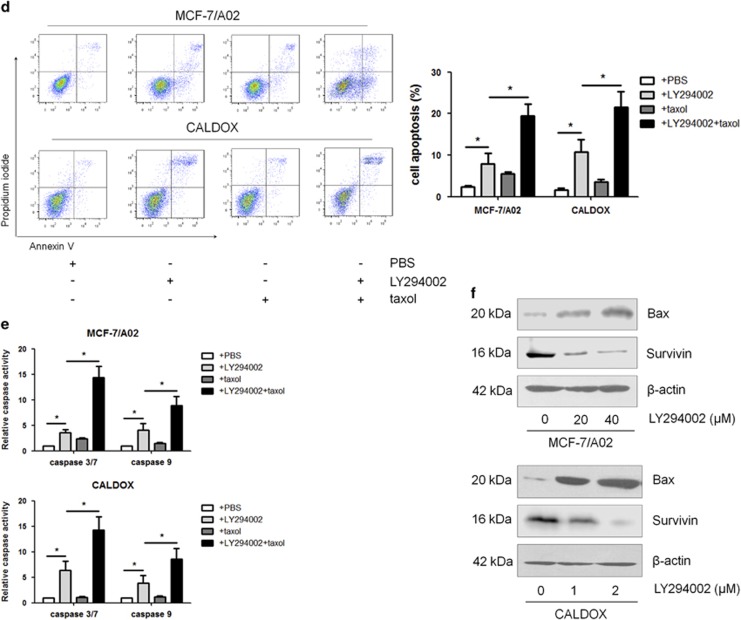
Exogenous VEGFA/FGF2 attenuates the effect of drug-sensitivity restoration of miR-205 through activating PI3K/AKT signaling in breast cancer cells. (**a**–**c**) MCF-7/A02 and CALDOX cells were stimulated with 30 ng/ml VEGFA or 20 ng/ml FGF2 for 72 h and treated by doxorubicin or taxol. (**a**) IC_50_ values of doxorubicin and taxol in MCF-7/A02-miR-205 cells (upper panels) and CALDOX-miR-205 cells (***lower panels***) in the presence or absence of VEGFA/FGF2. (**b**) Caspase-3/7 and caspase-9 activities in MCF-7/A02 (upper histograms) and CALDOX cells (lower histograms) after 48-h taxol treatment. (**c**) Expression levels of pAKT, AKT, Bax and Survivin in MCF-7/A02 and CALDOX with/without miR-205 overexpression in the presence or absence of VEGFA/FGF2 determined by western blotting. (**d** and **e**) Cells were treated with PI3K inhibitor LY294002 (10 *μ*M for MCF-7/A02 and 2 *μ*M for CALDOX) for 24 h and then treated by taxol (0.25 *μ*M for MCF-7/A02 and 2.5 nM for CALDOX) for another 48 h. (**d**) Annexin V/propidium iodide staining detected by flow cytometry after 48-h taxol treatment. Representative plots of three independent experiments are shown. Quantitative data show the average percentage of annexin V-positive cells (both in early apoptosis, lower right quadrants and late apoptosis, upper right quadrants) of three independent experiments (right panel). (**e**) Caspase-3/7 and caspase-9 activities in MCF-7/A02 (upper histograms) and CALDOX cells (lower histograms) after 48-h taxol treatment (**f**) Expression levels of Bax and Survivin were determined by western blotting in MCF-7/A02 and CALDOX after LY294002 treatment for 48 h. Numerical data represent mean±S.D. based on three independent experiments (*P<0.05), and the immunoblots are representative of three replicates

To further verify whether the phenotype observed after miR-205 overexpression was due to downregulation of VEGFA and FGF2, a rescue methodology was adopted. For this, MCF-7/A02-miR-205 and CALDOX-miR-205 cells were treated with doxorubicin or taxol in the presence or absence of VEGFA/FGF2. The addition of either VEGFA or FGF2 significantly increased the doxorubicin IC_50_ values of miR-205-overexpressing cells between 2.31- and 4.24-fold (Figure 4a). Equally, miR-205-overexpressing cells were more resistant to taxol in the presence of VEGFA/FGF2, the IC_50_ values increasing between 1.49- and 2.12-fold (Figure 4a and Supplementary Figure S3A). In agreement with the raised IC_50_ values, lowered caspase-3/7 and caspase-9 activities were observed in both miR-205-overexpressing drug-resistant cells in the presence of VEGFA/FGF2 after taxol treatment (Figure 4b). Moreover, the addition of VEGFA/FGF2 partially restored PI3K activity and survivin expression, which were abolished by miR-205 overexpression, and also decreased Bax expression, which was induced by miR-205 overexpression (Figure 4c). Thus the chemo-sensitizing effect of miR-205 in breast cancer depends on VEGFA/FGF2 downregulation.

To further confirm that PI3K activity is involved in the miR-205/VEGFA and FGF2 axis to regulate chemosensitivity of breast cancer cells, MCF-7/A02 and CALDOX cells were treated with LY294002, a well-characterized selective PI3K/AKT inhibitor. Similar to miR-205 overexpression, blocking PI3K/AKT signaling was accompanied by a resensitization of drug-resistant MCF-7/A02 and CALDOX cells to taxol, with an increment of apoptotic levels by 20% (Figure 4d). Measurement of caspase-3/7 and caspase-9 activities indicated an increase after taxol treatment in both drug-resistant cells when treated with LY294002 (Figure 4e). In addition, LY294002 treatment also enhanced Bax and reduced survivin expression (Figure 4f). Thus suppression of PI3K/AKT activity mimics the chemoresistant phenotype obtained by miR-205 overexpression.

### VEGFA and FGF2 protect breast cancer cells from cell death induced by chemotherapy

**Figure 5:**
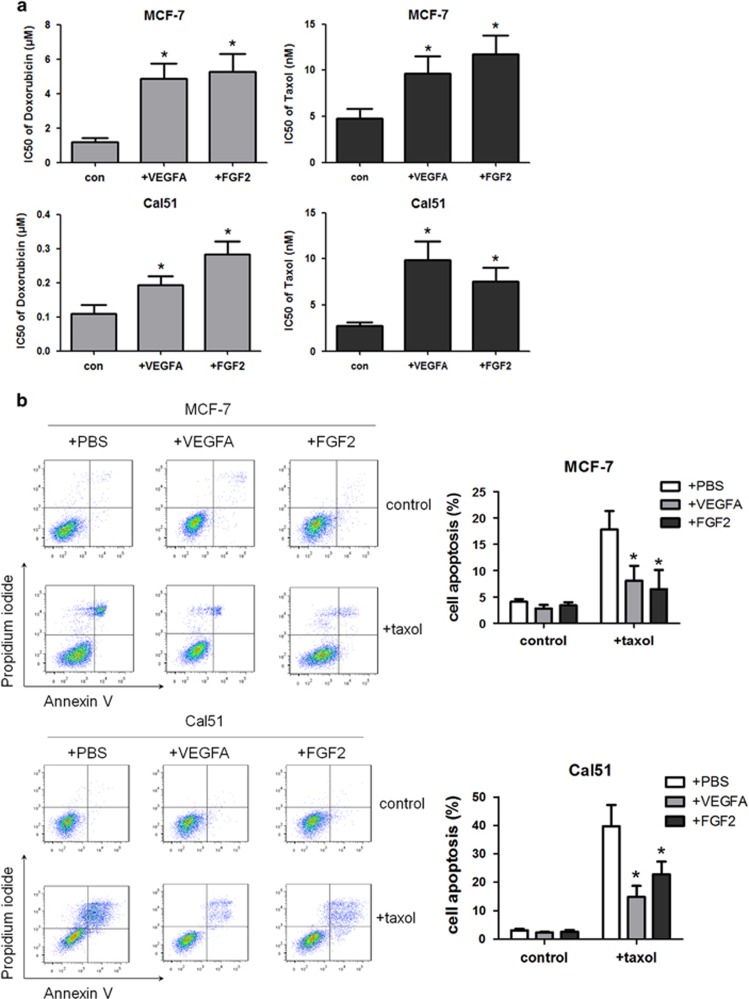

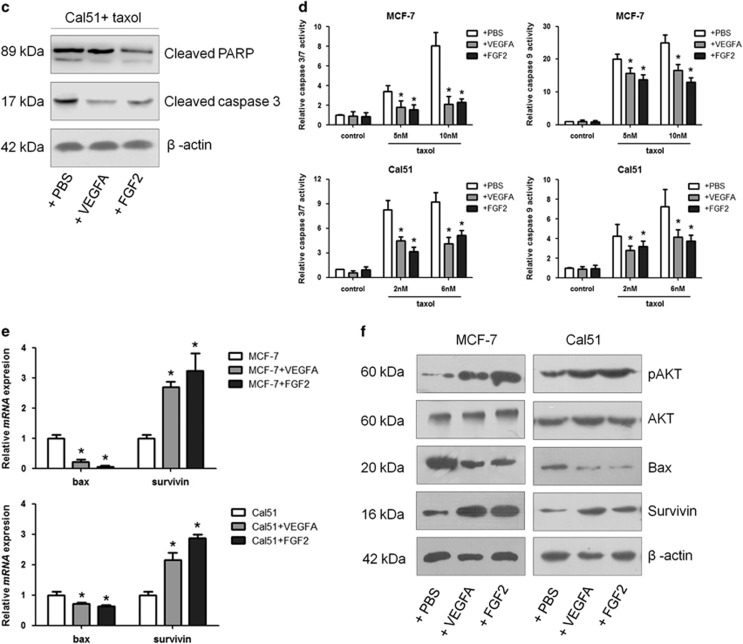
VEGFA and FGF2 protect breast cancer cells from chemotherapy-induced cell death through PI3K/AKT activation and apoptosis inhibition. MCF-7 and Cal51 cells were stimulated with 30 ng/ml VEGFA or 20 ng/ml FGF2 for 72 h and then treated by doxorubicin or taxol for 48 h. (**a**) IC_50_ values of doxorubicin and taxol in MCF-7 cells (upper panels) and Cal51 cells (lower panels) in the presence or absence of VEGFA/FGF2. (**b**) Annexin V/propidium iodide staining of cells determined by flow cytometry after 48-h taxol treatment (10 nM for MCF-7 and 2 nM for Cal51). Representative plots of three independent experiments are shown. Quantitative data show the average percentage of annexin V-positive cells (both in early apoptosis, lower right quadrants and late apoptosis, upper right quadrants) of three independent experiments (right panel). (**c**) Detection of cleaved PARP and cleaved caspase-3 by western blotting in Cal51 cell in the presence or absence of VEGFA/FGF2 after 2 nM taxol treatment for 48 h. (**d**) Caspase-3/7 and caspase-9 activities in MCF-7 and Cal51 cells after 48-h taxol treatment. (**e**) Expression levels of Bax and survivin in MCF-7 and Cal51 cells determined by qPCR in the presence or absence of VEGFA/FGF2. (**f**) Expression levels of pAKT, AKT, Bax and Survivin determined by western blotting in the presence or absence of VEGFA/FGF2. Numerical data represent mean±S.D. based on three independent experiments (*P<0.05), and the immunoblots are representative of three replicates

To further ascertain whether VEGFA and FGF2 are functionally important for chemoresistance, drug-naive MCF-7 and Cal51 cells were treated with doxorubicin or taxol, in the presence or absence of VEGFA/FGF2. The addition of either VEGFA or FGF2 significantly increased doxorubicin IC_50_ values in both MCF-7 and Cal51 cells between 1.75- and 4.45-fold (Figure 5a and Supplementary Figure S3B). Equally, drug-naive cells were more resistant to taxol in the presence of VEGFA/FGF2, the IC_50_ values increasing between 2.01-and 3.58-fold (Figure 5a and Supplementary Figure S3B). In agreement with these results, reduced apoptotic levels (Figure 5b), decreased cleavage of caspase-3 and PARP (Figure 5c) as well as lowered caspase-3/7 and caspase-9 activities (Figure 5d) were all observed in both MCF-7 and Cal51 cells in the presence of VEGFA/FGF2 after taxol treatment (cleaved caspase-3 and PARP were not determined in MCF-7 cells because of *CASP3* gene deletion). Moreover, the addition of VEGFA/FGF2 enhanced the levels of phosphorylated-AKT and survivin expression but downregulated Bax expression (Figures 5e and f). Thus exogenous VEGFA/FGF2 confers resistance to chemotherapeutics through enhancing PI3K/AKT signaling and inhibiting cell apoptosis in breast cancer cells.

### miR-205 upregulation inhibits tumor growth and enhances breast cancer chemosensitivity to doxorubicin and taxol *in vivo*

**Figure 6:**
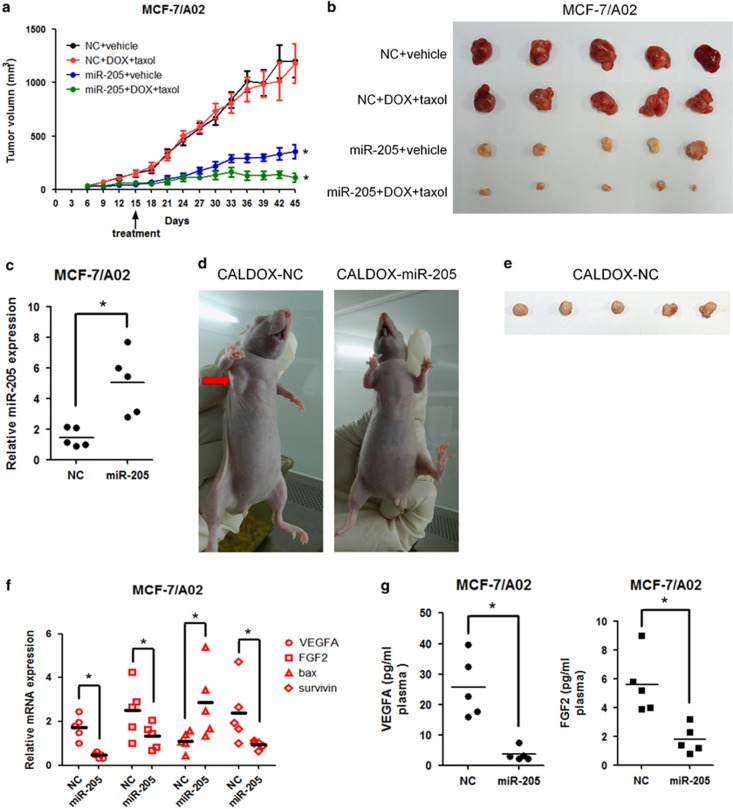
MiR-205 impairs the growth of breast cancer xenografts and increases chemosensitivity *in vivo*. Drug-resistant breast cancer cells stably expressing miR-205 or vector control (NC) were subcutaneously injected into the fat pad of nude mice (five per group). Fourteen days after implantation, mice were randomly split into two groups and treated with either vehicle or doxorubicin (1 mg/kg) plus taxol (2.5 mg/kg) every 3 days until they were killed 45 days after implantation, when xenografts were removed. (**a**) Size of MCF-7/A02 xenograft tumors after treatment with saline or drugs. Data are mean of tumor volume±S.D. of five tumors per group. (**b**) Image of tumors from control or miR-205-overexpressing MCF-7/A02 xenografts harvested at end point. (**c**) RNA was isolated from tumors and miR-205 was determined by qPCR. The individual tumor expression data (dots) and the mean values (line) are indicated. (**d**) Images of representative mice from control and miR-205-overexpressing CALDOX xenograft models. Arrow indicates the tumor developed only in the control group. (**e**) Image of tumors from control CALDOX xenografts harvested at day 28 after implantation. (**f**) RNA was isolated from tumors, and bax, survivin, VEGFA and FGF2 mRNA expression levels were determined by qPCR. The individual tumor expression data (dots) and the mean values (line) are indicated. (**g**) Expression levels of VEGFA and FGF2 determined by ELISA in plasma harvested at end point. The individual tumor expression data (dots) and the mean values (line) are indicated. *P<0.05

The regulatory role of miR-205 on drug sensitivity was further explored using a mouse xenograft model. For this, MCF-7/A02-miR-NC, MCF-7/A02-miR-205, CALDOX-miR-NC and CALDOX-miR-205 cells were injected into the mammary fat pad of nude mice. In the MCF-7 group, tumors overexpressing miR-205 reached approximately 300 mm^3^ at day 45 postimplantation, whereas tumors increased to approximately 1250 mm^3^ in the control group (Figures 6a–c). Remarkably, tumors generated with CALDOX cells overexpressing miR-205 failed to engraft, despite the fact that the controls reached approximately 300 mm^3^ 4 weeks postimplantation (Figures 6d and e).

Chemotherapeutic treatment using a combination of doxorubicin and taxol in the MCF-7/A02-miR-NC and MCF-7/A02-miR-205 mouse groups started at day 15 and was repeated every 3 days. As expected, the tumor continued to grow in the MCF-7/A02-miR-NC group, whereas the chemotherapeutic treatment was much more effective in tumors derived from MCF-7/A02-miR-205 cells (Figures 6a and b). Furthermore, consistent with *in vitro* analyses, tumors generated from miR-205-overexpressed cells had higher expression of Bax, as well as lower expression of survivin, than those generated with control cells (Figure 6f). miR-205-overexpressing tumors also showed lower human *VEGFA* and *FGF2* mRNA levels, and this was further confirmed at the protein level when human VEGFA and FGF2 plasma concentrations were determined (Figure 6g). Altogether, these data strongly support a tumor-suppressor role and promoter of chemosensitivity for miR-205 by repression of VEGFA and FGF2 and enhancing cell apoptosis in breast cancer.

## Discussion

NAC has become the standard treatment of locally advanced breast cancer as it can downstage the disease and improve surgery options.^39, 40^ Increasing efforts to identify patients that are sensitive or resistant to NAC treatment have highlighted the need for applicable biomarkers to predict response to NAC treatment. In this study, we show that miR-205 expression levels might be useful for predicting response to TAC regimen as NAC treatment in breast cancer patients. High miR-205 expression in breast cancer tissues strongly associates with increased sensitivity to TAC regimen, which is corroborated by other reports indicating an association between high miR-205 levels and better distant relapse-free survival in breast cancer patients.^32, 41^

It is well established that miRNAs can function as tumor suppressors or oncogenes depending on the cellular context and cancer types.^42, 43^ miR-205 is located in chromosome 1q32.2 and its expression pattern mirrors that of the miR-200 family.^44, 45^ miR-205 is upregulated in the progenitor subpopulation of a mouse mammary epithelial cell line, thus promoting cell proliferation and tumor initiation through targeting PTEN.^46^ However, increasing evidence shows downregulation of miR-205 in metastatic breast cancer cell lines, triple-negative breast cancer and metastatic breast secondary tumors, suggesting a tumor-suppressor role by reducing cellular proliferation, tumor angiogenesis and metastasis.^32, 34, 47, 48^ In addition, miR-205 has been found to modulate chemosensitivity in several human cancers but has opposite roles through targeting different genes. In prostate cancer, reduced expression of E-cadherin leads to docetaxel insensitivity, which is partially mediated by reduced miR-205 expression.^49^ miR-205 also enhances cisplatin toxicity in castration-resistant prostate cancer cells by targeting RAB27A and LAMP3,^50^ whereas in lung cancer, miR-205 overexpression is associated with carboplatin insensitivity by altering the expression of apoptosis-related genes.^51^ However, the effect of miR-205 in breast cancer chemoresistance is still poorly understood. Here, using chemoresistant breast cancer cell models, we show that miR-205 targets *VEGFA* as well as *FGF2* mRNA 3′-UTR and represses their expression, leading to impaired PI3K/AKT signaling and increased apoptosis both *in vitro* and *in vivo*. These findings are further supported by clinical data indicating that breast cancer patients with low VEGFA/FGF2 levels tend to achieve better response to NAC treatment (Figure 3f). Therefore, our study reveals a novel mechanism for miR-205 as a tumor suppressor and regulator of chemotherapy response.

miRNAs bind the 3′-UTR of target mRNAs repressing their translation. As binding to target sequences is not very stringent, miRNAs have a plethora of potential targets and it is plausible that additional molecules, other than VEGFA and FGF2, may be involved in mediating miR-205 chemo-sensitizing effect. Although we have ruled out ZEB-1 as the target gene of miR-205 in these two drug-resistant cell lines *in vitro*, it is unexpected that miR-205 overexpression in CALDOX cells leads to the failure of engraftment *in vivo*. This suggests that miR-205 may also be suppressing breast cancer cells' proliferation or tumorigenesis via additional mechanisms in line with our data (Figure 2c and Supplementary Figure S1B) and those found in melanoma^52^ and osteosarcoma.^24^

Although VEGFA and FGF2 were initially discovered as two of the most potent regulators of angiogenesis,^53^ it has become apparent that the function of VEGFA and FGF2 is not only limited to angiogenesis and vascular permeability but also includes embryogenesis tumor survival, proliferation and migration.^54, 55, 56^ Moreover, accumulating evidence demonstrates that VEGFA and FGF2 contribute to cytoprotection against drug toxicity via regulating apoptotic mediators and PI3K/AKT signaling pathway and thus has an important role in cancer drug resistance.^37^ For instance, loss of VEGFA expression increases sensitivity to 5-fluorouracil in colorectal cancer by inducing apoptosis.^57^ Beside promoting resistance to imatinib in chronic myeloid leukemia^36^ and promoting resistance to gefitinib in non-small cell lung cancer,^35^ FGF2 expression is also a stronger predictor of paclitaxel resistance than P-gp, p53 or Bcl-2 in several cancers.^58^ Several VEGFA-/FGF2-targeting miRNAs have been described in different cancers, including miR-503 in prostate cancer,^59^ miR-497 in hepatocellular carcinoma^60^ and miR-185 in clear cell renal cell carcinoma.^61^ Here we present data indicating that miR-205 targets VEGFA and FGF2 in breast cancer cells leading to decreased activity of PI3K/AKT signaling pathway, increased apoptosis and restoration of drug sensitivity in drug-resistant breast cancer cells.

In conclusion, our data demonstrate that elevated miR-205 expression levels enhance sensitivity to TAC chemotherapy in breast cancer patients. In addition, miR-205-mediated chemosensitivity is, at least in part, mediated by downregulation of VEGFA, FGF2 and subsequent increase of cell apoptosis. The efficacy of miR-205 in sensitizing breast cancer to chemotherapy may also support its application as a potential chemo-sensitizing agent. Our studies provide the rationale for using miR-205 as predictive biomarker to stratify patients before receiving TAC regimen as NAC and developing miR-205-based therapeutic agents for breast cancer treatment.

## Materials and Methods

### Patient characteristic, evaluation of treatment response and sample collection

Clinical data from 30 breast cancer patients who underwent neoadjuvant treatment from June 2014 to June 2015 are presented in Table 1. All patients received a TAC regimen of doxorubicin (50 mg/m^2^, q21d), docetaxol (75 mg/m^2^, q21d) and cyclophosphamide (500 mg/m^2^, q21d) on day 1 after undergoing core needle biopsies of their primary tumors and determination of the estrogen receptor (ER), progesterone receptor (PR) and Her2 status by immunohistochemistry. Treatment was continued in the absence of unacceptable toxicity for six cycles given every 21 days. This study was approved by the Medical Ethics Committee of Tianjin Medical University Cancer Institute and Hospital, and all the participants signed written informed consent forms. Because of the small patient cohort, NAC response rate was calculated as a continuous variable by determination of the change in the greatest tumor diameter following the formula (1−greatest tumor diameter after NAC/greatest tumor diameter before NAC) × 100%. Tumor tissue was obtained by a core biopsy prior to neoadjuvant treatment and immediately stored at liquid nitrogen. Total RNA was extracted using the miRCURY RNA Isolation Kit (Exiqon, Vedbaek, Denmark) following the manufacturer's instructions.

### Cells and drugs

Human breast cancer cell line MCF-7 and its MDR counterpart MCF-7/A02, human leukemia cell line K562, HL60, BJAB and their MDR counterparts were gifts from Professor Dongsheng Xiong (Institute of Hematology, PUMC, Tianjin, China) and were cultured as previously described.^21, 22^ Human breast cancer cell line Cal51, MTMEC and their MDR counterparts CALDOX and MD60 were cultured as previously described.^17, 20^ Doxorubicin and taxol were from Sigma (St Louis, MO, USA), VEGFA and FGF2 from R&D Systems (Oxon, UK) and PI3K inhibitor LY294002 from Cell Signaling Technology (Danvers, MA, USA).

### Plasmids, oligonucleotides and cell transfection

Lentivirus particles carrying miR-205 cloned into pGLV2-U6-Puro and packaging plasmid mix were purchased from GenePharma (Shanghai, China). MCF-7/A02 and CALDOX cells were infected with vector control (NC) or miR-205-overexpression virus and selected with puromycin as previously described.^23^ Plasmids (pMir-Target) carrying *VEGFA* 3′-UTR or *FGF2* 3′-UTR downstream from a luciferase gene were purchased from Life Technologies (Grand Island, NY, USA). phRGTK (Promega, Madison, WI, USA), expressing Renilla luciferase, was used to normalize for transfection efficiency essentially as described.^23^

### Cell viability analysis

MTT (3-(4, 5-dimethylthiazol-2-yl) -2, 5-diphenyltetrazolium bromide) assays were performed to evaluate the cell growth inhibitory effect in response to drug treatments and were used to determine the concentration of drug that inhibited cell growth by 50% (IC_50_) after 3 days of treatment.^31^

### Drug resistance clonogenic assay

Cells (1 × 10^5^ per well of a six-well plate) were treated with a single dose of doxorubicin or taxol for 1 week. Resistant clones were fixed with 4% paraformaldehyde and stained with 0.2% crystal violet and counted. Crystal violet retained in the cells was quantified by solubilization with 0.5% acetic acid and measurement of optical density at 592 nm.^31^

### Gene expression analysis

RNA isolation, reverse transcription and real-time qPCR were performed as previously described^23^ using specific primers for each gene (Supplementary Table S3). TaqMan miR-205 and RNU6 RNA (used as a normalizer) assays (Life Technologies) were used for miR-205 detection.^23^

### Antibodies

Antibodies for immunodetection following immunoblotting procedures^23^ were phospho-AKT (D9E), AKT1 (C73H10), Bax (D2E11), cleaved PARP (D64E10), cleaved caspase 3 (5A1E) (Cell Signalling Technology), Survivin (ab76424, Abcam, Cambridge, UK) and *β*-actin (sc47778, Santa Cruz Biotechnology, Dallas, TX, USA). Appropriate peroxidase-conjugated secondary antibody (Cell Signaling Technology) were detected by enhanced chemiluminiscence.^23^

### Annexin V staining

Cell apoptosis was measured by flow cytometry using an annexin V-FITC apoptosis detection kit (Becton–Dickinson, San Diego, CA, USA) essentially as described.^31^

### Caspase activity

Cells (1 × 10^4^) were incubated with taxol and/or LY294002 in a 96-well plate for 24 h. Caspase-3/7 and caspase-9 activities were measured using Caspase-Glo 3/7 Assay and Caspase-Glo 9 Assay Kits, respectively (Promega) following the protocol recommended by the manufacturer.

### Enzyme-linked immunosorbent assay (ELISA)

Cells (3 × 10^5^) were plated in a six-well plate with DMEM medium supplemented with 10% serum and incubated for 24 h. Medium was replaced with 2 ml serum-free DMEM for another 24 h and then collected for ELISA. Blood was taken from tumor-bearing mice and immediately placed into EDTA-treated tubes (BD Biosciences, San Jose, CA, USA). Plasma was obtained by removing blood cells through centrifugation at 1800 × *g* for 15 min. VEGFA and FGF2 levels in both the cell culture medium and plasma of mice were measured by commercial VEGFA and FGF2 ELISA Kit (R&D Systems) according to the manufacturer's instructions. This kit uses antibodies to recognize human VEGFA and FGF2 with no cross-reactivity with the mouse corresponding proteins.

### *In vivo* tumor assay

Cells (5 × 10^5^) were resuspended in a total volume of 100 *μ*l containing 50% Matrigel (BD Biosciences) in PBS and injected into the mammary fat pad of 4–5-week-old nude mice. Tumor sizes were measured every 3 days in two dimensions using a caliper, and the tumor volume was calculated with the following formula: tumor volume (mm^3^)=0.5 × *ab*^2^ (*a* and *b* being the longest and the shortest diameters of the tumor, respectively). Fourteen days after cell injection, tumor-bearing mice were randomly divided into different groups (five animals per group) for doxorubicin (1 mg/kg) plus taxol (2.5 mg/kg) treatment or control (saline). Drug was injected every 3 days, and the tumor volume was monitored until the mice were killed in a humane manner. Tumors were collected, RNA was isolated, using a mirVana miRNA Isolation Kit (Life Technologies) following the manufacturer's protocol, and gene expression analysis was performed by qPCR. All animal studies were performed at the National Institutes of Health (Tianjin Cancer Hospital) in accordance with guidelines under Institutional Animal Care and Use Committee (IACUC) and approved by the Committee on the Ethics of Animal Experiments of the Tianjin Cancer Hospital.

### Statistical analyses

Statistical analysis was carried out by one-way ANOVA, and comparisons among groups were carried out by the independent sample two-sided Student's *t*-test. Spearman rank test was used to identify the correlation between miR-205/VEGFA/FGF2 and NAC response rate, as well as the correlation between miR-205 and VEGFA/FGF2. All statistical analyses were performed using the SPSS 22.0 software (SPSS Inc., Chicago, IL, USA). *P*-value <0.05 was considered as statistically significant.

## Figures and Tables

**Table 1:** Clinical information of the 30 patients included in the study

**Characteristics**	**Number of patients**	**%**
*Age 53 (27–67) years*		
≤35	2	6.7
35–55	16	53.3
>55	12	40.0
		
*Histology*		
Infiltrating ductal carcinoma	12	40.0
Infiltrating (mixed) carcinoma	14	46.7
Others	4	13.3
		
*TNM stage*		
II	7	23.3
IIIA	19	63.3
IIIB–IV	4	13.3
		
*Estrogen receptor status*		
Negative	13	43.3
Positive	17	56.7
		
*Progesterone receptor status*		
Negative	20	66.7
Positive	10	33.3
		
*Her2 status*		
Negative	19	63.3
Positive	11	36.7

## Abbreviations

NAC: neoadjuvant chemotherapy
miRNA: microRNA
EMT: epithelial–mesenchymal transition
TAC: docetaxol, doxorubicin and cyclophosphamide
VEGFA: vascular endothelial growth factor A
FGF2: fibroblast grow factor-2
PI3K: phosphatidylinositol 3-kinase
RT-qPCR: reverse transcription quantitative real-time PCR
MDR: multidrug resistance
