# Supplementary material for: miRNA-205 targets VEGFA and FGF2 and regulates resistance to chemotherapeutics in breast cancer
Source: Cell Death Dis. 2016 Jun 30;7(6):e2291–. doi: 10.1038/cddis.2016.194 (PMC5108343; doi:10.1038/cddis.2016.194)
Supplement: Supplementary Information [file cddis2016194x1.docx]

**Supplementary Information**

**miRNA-205 targets VEGFA and FGF2 and regulates resistance to chemotherapeutics in breast cancer**

*Yunhui Hu^1,2 †^, Yufan Qiu^1^, Ernesto Yagüe^3^, Wei Ji^2^, Jingjing Liu^1^ and Jin Zhang^1,2 †^*

*^1^* *The 3^rd^ Department of Breast Cancer, China Tianjin Breast Cancer Prevention, Treatment and Research center,* *Tianjin Medical University Cancer Institute and Hospital, National Clinical Research Center of Cancer,* *Huan Hu Xi road, Ti Yuan Bei, He xi district, Tianjin, 300060, PR China*

*^2^ Key laboratory of breast cancer prevention and therapy of ministry of education, Huan Hu Xi road, Ti Yuan Bei, He xi district, Tianjin, 300060, PR China*

*^3^ Cancer Research Center, Division of Cancer, Faculty of Medicine, Imperial College London, Hammersmith Hospital Campus, London W12 0NN, Great Britain*

*^†^ Senior corresponding authors contributed equally*

*Jin Zhang: The 3^rd^ Department of Breast Cancer, Tianjin Medical University Cancer Institute and Hospital, Huan-Hu-Xi road,Ti-Yuan-Bei,He xi district, Tianjin, 300060, PR China. Tel: +86-22-23340123; Email:* [*zhangjin@tjmuch.com*](mailto:zhangjin@tjmuch.com)

*Yunhui Hu: The 3^rd^ Department of Breast Cancer, Tianjin Medical University Cancer Institute and Hospital, Huan-Hu-Xi road,Ti-Yuan-Bei,He xi district, Tianjin, 300060, PR China. Tel: +86-13702046550; Email:* [*yunhuihu200408@163.com*](mailto:yunhuihu200408@163.com)


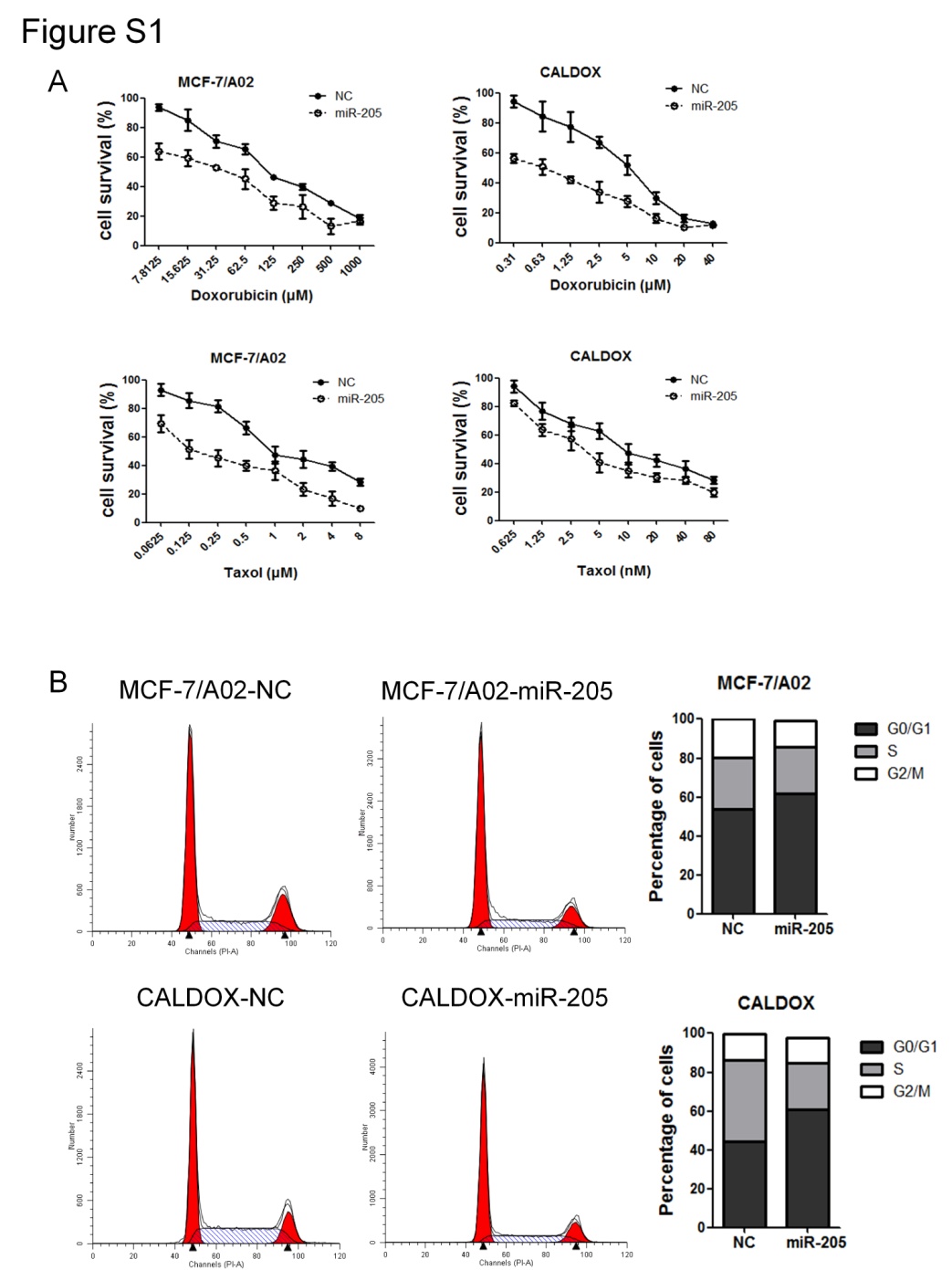


Figure S1. (A) Dose-response curves used to calculate the IC_50_ of doxorubicin and taxol for MCF-7/A02 (*left panels*) and CALDOX (*right panels*). (B) Cell cycle profiling by flow cytometry. Cells were stained with propidium iodide and gated according to their fluorescence to differentiate cell cycle phases (from left to right: G0/G1, S, G2/M). Experiments were performed in triplicate and representative plots are shown (*left panels*). Histogram data (*right panels*) represent the average of three independent experiments. For clarity error bars have been omitted (typical variation between experiments: ± 5%)


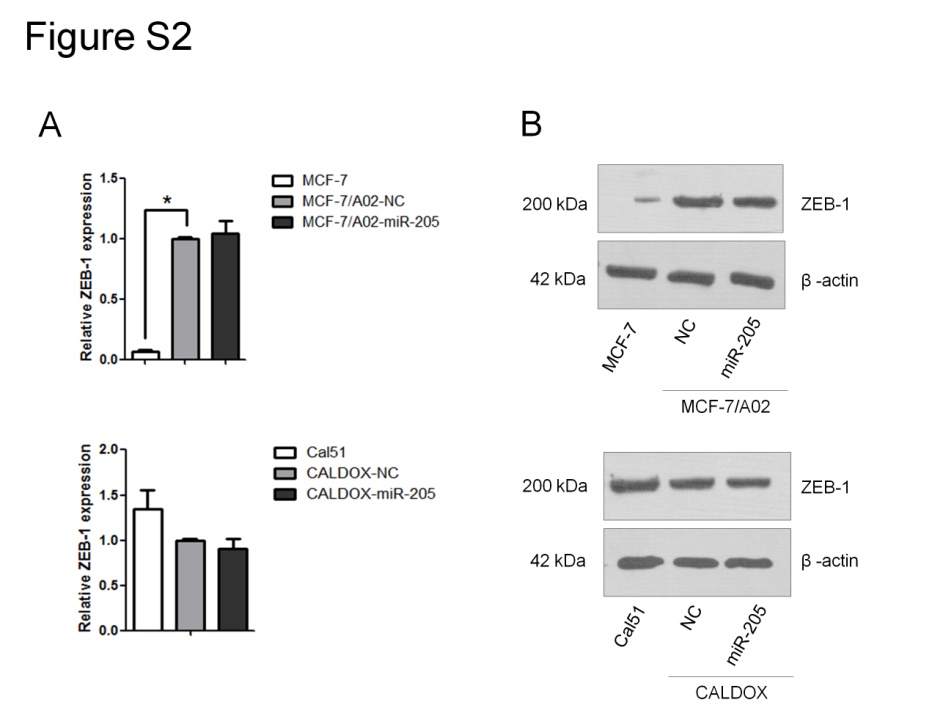


Figure S2. Expression levels of ZEB-1 in mir-205-transfected drug-resistant cells determined by qPCR (A) and western blotting (B). Numerical data represent mean ± SD based on three independent experiments (**P* < 0.05) and the immunoblots are representative of three replicates.


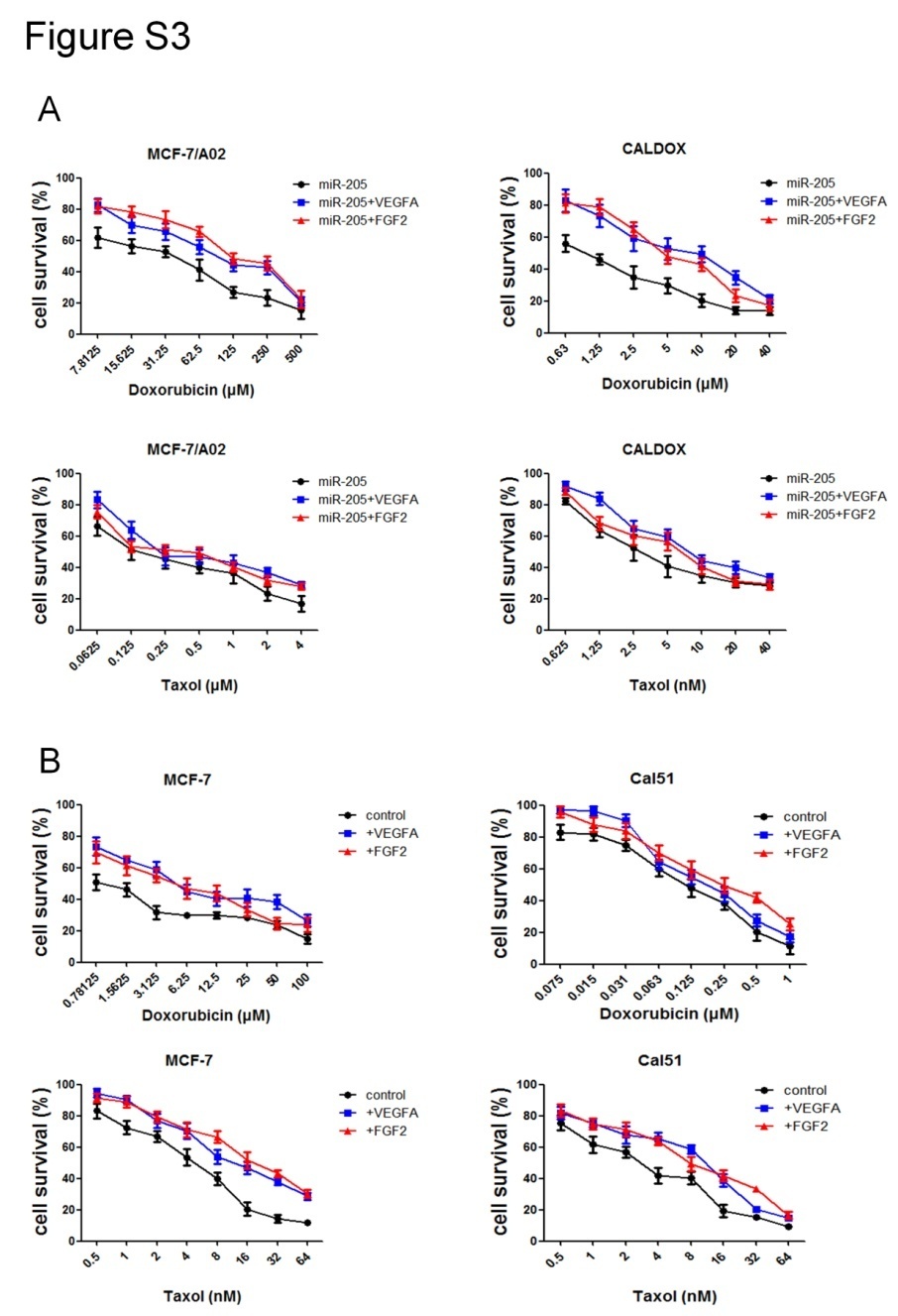


Figure S3. (A) Dose-response curves used to calculate the IC_50_ of doxorubicin and taxol for MCF-7/A02-miR-205 cells (*left panels*) and CALDOX-miR-205 cells (*right panels*) in the presence or absence of VEGFA/FGF2. (B) Dose-response curves used to calculate the IC_50_ of doxorubicin and taxol for MCF-7 cells (*left panels*) and Cal51 cells (*right panels*) in the presence or absence of VEGFA/FGF2

**Table S1** Drug sensitivity in MCF-7/A02 cells

| Drug | IC_50_ | | MCF-7/A02 resistance ratio |
| --- | --- | --- | --- |
|  | MCF-7 | MCF-7/A02 |  |
| Doxorubicin | 2.64 μM | 128.3 μM | 48.6 |
| Etoposide | 5.29 μM | 206.8 μM | 39.1 |
| Taxol | 4.77 nM | >500 nM | >100 |
| Mitoxantrone | 8.7 μM | 161.8 μM | 18.6 |

**Table S2** Drug sensitivity in CALDOX cells

| Drug | IC_50_ | | CALDOX  resistance ratio |
| --- | --- | --- | --- |
|  | Cal51 | CALDOX |  |
| Doxorubicin | 0.14 μM | 5.73 μM | 40.9 |
| Etoposide | 0.78 μM | 26.8 μM | 34.4 |
| Taxol | 2.77 nM | 8.68 nM | 3.13 |
| Mitoxantrone | 2.49 nM | >300 nM | >120 |

**Table S3** Oligonucleotides used for real-time PCR

| Name | Sequence (5' to 3') |
| --- | --- |
| RPS14-forward | TCACCGCCCTACACATCAAACT |
| RPS14-reverse | CTGCGAGTGCTGTCAGAGG |
| bax-forward | CGAGTGGCAGCTGACATGTTTT |
| bax-reverse | TGAGGCAGGTGAATCGCTTGAA |
| survivin-forward | GGACCACCGCATCTCTACAT |
| survivin-reverse | GACAGAAAGGAAAGCGCAAC |
| VEGFA-forward | CCAGCAGAAAGAGGAAAGAGGTAG |
| VEGFA-reverse | CCCCAAAAGCAGGTCACTCAC |
| FGF2-forward | CTGGCTATGAAGGAAGATGGA |
| FGF2-reverse | TGCCCAGTTCGTTTCAGTG |
